# The elephant in the room: critical reflections on mortality rates among individuals with Parkinson’s disease

**DOI:** 10.1038/s41531-023-00588-9

**Published:** 2023-10-19

**Authors:** Lisanne J. Dommershuijsen, Sirwan K. L. Darweesh, Yoav Ben-Shlomo, Benzi M. Kluger, Bastiaan R. Bloem

**Affiliations:** 1 Center of Expertise for Parkinson and Movement Disorders, Department of Neurology, Donders Institute for Brain, Cognition and Behaviour, Radboud University Medical Center, Nijmegen, The Netherlands; 2 Population Health Sciences, Bristol Medical School, University of Bristol, Bristol, UK; 3 Departments of Neurology and Medicine, University of Rochester Medical Center, Rochester, NY USA

**Keywords:** Parkinson's disease, Epidemiology

## Abstract

In our efforts to create more public awareness about Parkinson’s disease, we often emphasize the tremendous impact of this common disease on an individual’s life. However, in public awareness campaigns, we largely avoid discussions on the survival of people with Parkinson’s disease (PwP). Many clinicians even state that the survival with Parkinson’s disease is close to normal. In this article, we contemplate on findings regarding the mortality of Parkinson’s disease in order to spark a discussion about what information we should provide to affected individuals and their near ones about the life expectancy of PwP. Our narrative review of the evidence indicates that although the survival of PwP has improved over time, PwP still live fewer years than their age- and sex-matched population comparators, albeit at older ages this difference can be small. We feel that it is important to emphasize this information towards PwP, the general public, policymakers and funding bodies. We hope that this will help to create a better understanding of the enormous impact that this disorder can have on affected individuals, even beyond the disability that is experienced during life.

## Background

Parkinson’s disease (PD) is the fastest-growing and second most common neurodegenerative disease worldwide^1–3^. PD has a tremendous impact on the quality of life of people living with PD (PwP) and their near ones. In our efforts to create more public awareness about this devastating neurodegenerative disease or when seeking research funding, we often tend to emphasize the many negative aspects of PD. Anecdotally, discussions on the mortality of PD are largely avoided. Moreover, when counselling PwP and their near ones about the prognosis of PD, our experience is that neurologists often inform their patients that the survival with PD is close to normal. We appreciate that the tone of voice may depend on the context at hand, but which rigorous facts should inform the debate? The objective of this article is to spark a discussion about how to inform PwP and their near ones about the mortality of PD and how to discuss this topic in public awareness campaigns. We aim to break the taboo around mortality in PD in order to improve patient care and make way for advance care planning.

## Mortality of Parkinson’s disease

The mortality of PD can be qualified both with relative and absolute risk measures (explained in Box 1). Most of our data on mortality risks come from cohort studies, the qualitatively best ones having included newly diagnosed PwP. Such studies have shown that PwP on average have a 50% relatively increased mortality compared to a reference population^4^. The relative increase in mortality is higher for atypical forms of parkinsonism, such as multiple system atrophy or dementia with Lewy bodies^5^. However, relative estimates are difficult to translate meaningfully when providing counselling to PwP. An obvious question that arises with these estimates is what is the most appropriate “comparison population”. Furthermore, group-level mortality estimates can mask marked variability and do not provide the most relevant information to an individual living with PD. For such individualized prognostication of survival, the creation of externally validated prognostic models is needed^6^. However, such models are scarce in the current literature. An alternative population-based strategy that can be better applied to most estimates available in the literature is to stratify mortality risk into relevant sub-groups, either based on socio-demographic factors, e.g. age groups, or clinical factors.

**Fig. 1:**
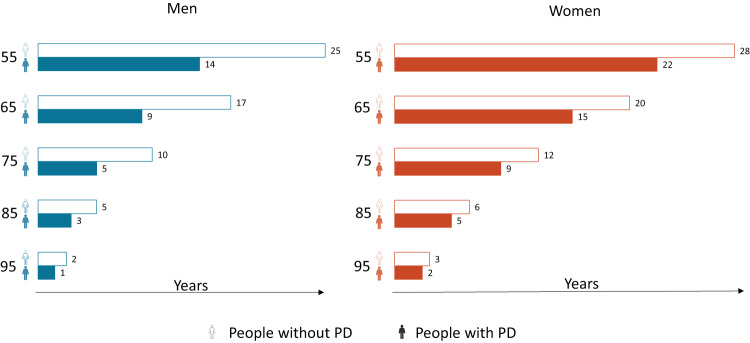
The life expectancy of people with Parkinson’s disease visualized. The bars show the different life expectancies for men and women, per 10-year age band from 55 years and for PwP and people without PD. These data can also be used to derive the average years of life lost due to PD. Life expectancy estimates include the unweighted sex-specific average of Ishihara et al. (2007), Hobson et al. (2010) and Dommershuijsen et al. (2020)^8–10^. The results of Ishihara et al. (2007) are based on visual estimates from Figure 2 in that article. The individual studies showed variable estimates with wide confidence intervals. A formal meta-analysis remains necessary. The remaining life expectancy is dependent on individual characteristics and thus uncertainty regarding the individual prognosis should be emphasized when communicating this to patients. This visualisation needs patients’ testing before being implemented in clinical practice. PD Parkinson’s disease.

Most people find visual representations of risk more easy to comprehend than numerical tabulations^7^. One approach we feel is helpful is illustrated in Fig. 1, which presents estimated average life expectancies at different ages^8–10^. What can be clearly seen in this figure is that life expectancy reduces with increasing age and that, in every age group, women live longer than men. This is similar to longevity in the general population^11^. An important factor explaining the life expectancy difference between men and women includes the higher cardiovascular mortality in men^12^. More specifically to PD, an explanation of the sex difference in life expectancy might be that men have been more commonly exposed to environmental risk factors due to professional risks (e.g., pesticides and heavy metals), which are associated with shorter survival^13,14^. An extensive overview of possible explanations of sex differences in PD has previously been published elsewhere^15,16^. Fig. 1 also shows that PwP live fewer years than people without PD. According to the non-weighted average life expectancy of three studies shown in this figure, 55 year old men would lose almost half of their remaining life expectancy when diagnosed with PD and women of the same age would lose about 20% of their remaining life expectancy. Importantly, the studies included in this unweighted average showed variable life expectancy estimates and confidence intervals overlapped at higher ages. A formal meta-analysis should be performed to confirm these numbers. Finally, Fig. 1 shows that the difference in life expectancy between PwP and people without PD becomes smaller with age. The impact of PD on mortality is thus not similar for all age groups; an estimated difference in life expectancy of just one year, with overlapping confidence intervals, is all that remains for the oldest subgroup. The observation that earlier disease onset results in the greatest reductions in life expectancy makes intuitive sense, as the median survival with PD is approximately 10 years^4^ and PwP who are considerably older at the time of diagnosis will often not live that long and thus “die with, but not from PD” due to other competing causes of death.

Box 1 Relative versus absolute risks Epidemiologists quantify risk using two complementary approaches. The most common measure is known as a relative effect estimate (e.g. relative risk, relative rate, hazard ratio) and is a unitless ratio. This is derived by comparing the risk of an exposed group (PwP) to an unexposed group (people without PD) and dividing one by the other. Hence, no difference is indicated by the value of one and greater values indicate an increased risk, whilst values less than one indicate a decreased risk. This approach is very helpful in trying to measure the causal impact of an exposure. Another more public health and policy relevant approach is to use the absolute risk difference, which is simply the risk in the exposed group minus the risk in the unexposed group. This absolute risk difference reframes the relative effect and, importantly, has units which help to contextualise the effect.

For example, relative estimates can make for headline grabbing statements such as exposure to “X” doubles (relative risk = 2) your risk of disease. But if this exposure merely increases the risk from 2 per 100,000 to 4 per 100,000 (risk difference = 2 per 100,000) there is no need for the public to be overly concerned.

## Causes of death in people with Parkinson’s disease

The empirical evidence highlights a reduced survival in PwP compared to age-matched peers, especially in PwP who develop dementia during the disease course^4^. Nevertheless, the question remains what causes this premature mortality? Studies have attempted to answer this question using data from death certificates. Such analyses have shown that around half of deceased PwP do not have PD being mentioned on their death certificate^17,18^. Whilst some of this may represent under-reporting, it is mostly because PwP also die from causes unrelated to PD^17,18^, e.g. cancer. Importantly, PD should be listed as the cause of death if it is contributory to death or if it causes a sequence of events leading to death, e.g. when a person dies from a hip fracture which is the result of a fall due to freezing of gait in advanced PD.

The most common primary cause of death of PwP is pneumonia^17,19^, which is often secondary to aspiration as a result of immobility and dysphagia. Other complications from PD such as injuries resulting from falls will also contribute to an increased mortality – hip fractures are particularly notorious in this regard^20^. These mortality patterns are mirrored by the reasons for emergency admissions in PwP: pneumonia, motor decline, urinary tract infections and falls have been described as the most common reasons for hospitalization in PwP^21^.

## Time trends in Parkinson’s disease mortality

The overall mortality from PD has increased vastly in the past couple of decades. Worldwide, the total number of deaths from PD has almost tripled between 1990 and 2016^1^. Explanations for this increase include ageing, better recognition of PD, changes in cause of death certifications and a decline in competing causes of death such as cardiovascular disease. An increasing incidence of PD could also contribute to this observed increase in mortality^2^, although the evidence base for this remains limited and controversial.

In the general population, decreased rates of cardiovascular deaths have contributed to improved life expectancies^22^. In PwP specifically, survival has also improved, but not as much as in reference populations of people without PD^23^. This discrepancy has resulted in an increased mortality gap between PwP and controls. Between 2007 and 2016, the decline in adjusted mortality rates per year in the group of PwP was calculated to be 1.2 per 1000 person-years, compared to a decline of 2.4 per 1000 person-years in the non-PD group^23^. The life expectancy of PwP would be expected to increase in future years, as we will discuss in more detail in the next paragraph. When the diagnosis is established at the age of 65, this increase is estimated to amount to 3 years in 20 years’ time^24^.

## Effect of treatment on Parkinson’s disease mortality

An increase in life expectancy of PwP might result from generic improvements in lifestyle and better healthcare overall, but also from better PD therapies. A range of observational studies performed at the end of the 20th century investigated the effect of the introduction of levodopa on the survival of PwP^25–27^. Some observational studies found an improvement in survival, but all these studies were at high risk of bias. There are no long-term randomised trials available given the clear benefits of dopaminergic drug treatment on motor and non-motor functioning^28^, on reducing the risk of institutionalization and on improving mobility. In general, reviews report little evidence that the introduction of dopaminergic drugs for PD influenced survival^4,29^ or delayed the onset of serious PD complications such as falls and dysphagia, which are major contributors to death in PwP^29^. Some studies have reported a survival benefit after Deep Brain Stimulation (DBS)^30,31^, especially with early treatment^31^, though not all^32,33^. These results are also at high risk of bias because of the strict inclusion criteria for DBS – operated patients may have been better overall than those who had been denied surgery – and because of the possibly more intensive follow-up after DBS, which could lead to a generally better management approach, and thereby better outcomes^30^.

Physical therapy is an important part of the symptomatic treatment of PD^34^. Exercising has been associated with smaller declines in motor and non-motor symptoms and health-related quality of life in observational studies^35,36^. Similarly, RCTs have shown positive effects of physical activity on stabilizing 6-month progression in motor symptoms and quality of life^37,38^. Additionally, positive effects have been described on neuroplasticity in both motor and cognitive brain networks involved in PD^39^. However, further evidence is required before concluding that exercise can be considered a long-term disease-modifying treatment^40^. Physical therapy – when delivered by specifically trained therapists – might even contribute to improved survival, possibly by preventing common complications of PD^34^. These putative beneficial effects may be mediated by both the generic benefits of physical activity (e.g. improved cardiovascular fitness) and by PD-specific benefits (e.g. a reduction in fall-related injuries due to gait training). More recent work suggests that speech-language therapy can help to prevent aspiration pneumonia in PwP^41^, but survival was not studied here. These benefits of allied health therapy can be amplified by referring PwP to specialized allied health therapists who have received a dedicated PD-specific training program according to evidence-based guidelines and who are experienced with a high caseload^34,41^.

Importantly, access to care for PD is not equitably distributed among PwP across the world^42^. There are important racial and ethnic disparities, both in making the diagnosis of PD as well as in the availability of treatments. In high-income countries, people from minority racial or ethnic groups are generally less likely to receive neurologic care^3,43,44^. Additionally, there are large inequalities in access to neurologic care in low- and middle-income countries^42^. Highly needed basic care for PwP is thus not available to all people in need, let alone DBS or specialized allied health therapies. This inequitable access to care will widen existing health disparities and negatively affect health outcomes, such as quality of life^45^ and survival^46^. In order to improve the prognosis and survival of PwP globally, we must increase healthcare professionals’ awareness of health disparities and better understand the underlying causes in order to develop interventions to reduce these disparities in PwP^3,42,43^.

## Need for advance care planning

Whilst it is a normal tendency to “sugar coat” the diagnosis of any chronic progressive disease, clinicians have the responsibility to present honest prognostic information and to communicate this information to patients in a sensitive and time-appropriate fashion. Providing PwP with reliable information about their prognosis is essential to initiate advance care planning (ACP), a dynamic process during which patients are supported in ensuring that healthcare is in line with their values, needs, preferences and goals^47,48^. Early palliative care, including ACP, can help to anticipate uncertainties regarding disease-related and end-of-life issues and may have important benefits for quality of life and patient and family satisfaction^49,50^. In current clinical practice, ACP is often postponed until relatively late in the course of PD^51^: Less than one in every ten neurologists initiates ACP at the time of PD diagnosis^52^. Although postponing these discussions until they are more imminent might be valued by some patients^53^, several studies have shown that most PwP appreciate their healthcare professional initiating ACP discussions early on in the disease course^47,53–55^. PwP and their near ones desire honest and accurate prognostic information, including the option for learning more about advanced disease and mortality^50,53,56^. Delaying conversations until “the right time” may mean a postponement to very late disease stages when many patients are no longer able to participate well due to communication issues, cognitive impairment, or a sudden incapacitating illness.

Preferably, patients’ values and readiness for ACP conversations are discussed early in the disease course and are repeated regularly (e.g. annually) as a part of standard care^48,50^. A roadmap that explains possible life changes due to PD and implications for decision-making can be helpful to guide shared decision-making practices^50^. In this way, best and/or worst case scenarios can be explored and more concrete plans can be made for the future. Tailored information provision remains essential given the heterogeneity of PD and the varying level of knowledge and desire for details of PwP and their families^50,53^.

## Implications for clinical care, education, public advocacy and research

In this article, we have raised the important question whether Parkinson’s disease is a deadly condition. Our narrative review of the evidence indicates that although the survival of PwP has improved over time, even today, PwP still live fewer years than their age- and sex-matched population comparators, albeit at older ages this difference can be small.

We can offer some guidance for the clinical consultation of PwP and their near ones. Healthcare professionals treating PwP should enquire about patients’ and their families’ preferences regarding the amount and type of prognostic information they are given and when they wish to have such discussions. We recommend that healthcare professionals inform PwP about the possible PD trajectory and the survival after diagnosis using a variety of methods that are best suited for each individual patient. In general, probabilities will be less comprehensible than absolute measures, such as the average years of life lost. Patients’ abilities to comprehend numerical data differ and various formats may be helpful, especially visual presentations that are interactive and can be modified to suit the patient’s individual circumstances. There are valuable lessons to be learnt from the wealth of information and misinformation that emerged during the recent COVID-19 pandemic^57^. When discussing prognostic information with PwP, it remains essential to emphasize the uncertainties regarding individual predictions.

Recognition that PD reduces survival will not only have a direct impact on the information provision to PwP, but it could also impact future research. This recognition for instance supports the inclusion of mortality as an outcome measure in long term randomized controlled trials or trials including PwP with more advanced disease. Several key gaps in knowledge around mortality of PD remain, which are described in Table 1.

**Table 1:** Key knowledge gaps regarding Parkinson’s disease mortality.

Theme	Objective
Age- and sex-specific case fatality rates	Stratified data on case fatality rates are necessary to establish simple figures to explain the prognosis of PD to PwP.
Absolute life expectancy	Large studies with a wide age range are required to provide reliable estimates of the absolute life expectancy of people with parkinsonism across subgroups defined by demographic and clinical characteristics, especially in people with atypical parkinsonism.
Uncertainty in diagnosis and prognosis	Better prediction models for the individual course of PD and the survival with PD are needed.
Causes of death	Immediate causes of death in PwP should be evaluated to be able to study the effect of secondary prevention interventions.
Influence treatment on prognosis	The influence of pharmacological and non-pharmacological interventions on long term PD survival should be studied further.
Health disparities in diagnosis and care	In order to develop interventions to reduce health disparities in PD, a better understanding of the causal mechanisms underlying these disparities is needed.
Advance care planning	Patient and healthcare provider related factors determining the appropriate timing of ACP discussions should be studied to develop strategies for shared-decision-making.
Patients’ needs and wishes upon diagnosis	Qualitative studies are required to ascertain how to provide prognostic information about PD in a patient friendly and culturally sensitive manner.

*PD* Parkinson’s disease, *PwP* People with Parkinson’s disease, *ACP* Advance care planning.

Finally, knowledge about the reduced survival with PD has implications for messaging and advocacy. Public advocacy and education must not soft sell the impact of PD, but should provide honest messaging about the survival with PD. Acknowledging the reduced survival of PD will create a greater sense of urgency for future research.
